# Fatal parvoviral myocarditis: A case report and review of literature

**DOI:** 10.1186/1746-1596-3-21

**Published:** 2008-04-30

**Authors:** Fabio Tavora, Luis F Gonzalez-Cuyar, Jay S Dalal, Michael T O'Malley, Richard Zhao, Hong Q Peng, Allen P Burke

**Affiliations:** 1 Department of Pathology, University of Maryland, Baltimore, MD, USA; 2 Department of Internal Medicine, University of Maryland, Baltimore, MD, USA; 3 Department of Molecular Diagnostics, University of Maryland, Baltimore, MD, USA

## Abstract

**Background:**

Histologically documented cases of parvoviral myocarditis are exceedingly rare.

**Case presentation:**

Here, we report a 41-year old African American immunocompetent patient who died of parvoviral myocarditis after a 10 day illness characterized by fever, headaches, generalized arthralgias, and a maculopapular rash.

Autopsy revealed an infiltrate myocarditis composed primarily of T-lymphocytes and macrophages associated with extensive myocardial fibrosis. The diagnosis of parvovirus was determined by polymerase chain reaction (PCR) on both pre-mortem serum and post-mortem myocardial tissue

**Methods:**

DNA was extracted from tissue and serum and primers were used to amplify DNAsequences of parvovirus B19 using nested polymerase chain reaction (PCR).

**Conclusion:**

The diagnosis of parvovirus should be considered in cases of fatal myocarditis, and diagnosis can be confirmed at autopsy by molecular techniques.

## Introduction

Parvovirus B19 (PB19) was discovered in 1974 and is the only member of the family *Parvoviridae *known to be pathogenic in humans[1]. It is endemic in humans worldwide, as antiparvovirus IgG antibody is present in asymptomatic patients in the United States, Europe, and Asia[2]. Infection is generally self-limited and in children typically presents as erythema infectiosum. Other manifestations of parvovirus infection include hydrops fetalis, arthropathy, and acute hepatitis [3-6]. In immunocompromised patients, PB19 infection of erythropoetic cells may result in anemia, and occasionally acute lung infections[7].

The most common etiologies are infectious agents, hypersensitivity responses, or immune-related injury[8]. However, the etiology might go unrecognized secondary to difficulties in identifying infectious agents. This is particularly truth for viruses in the early stage of infection before the virus is cleared by the immune system. The detection of viral genomes in endomyocardial biopsies by molecular techniques, especially PCR, has greatly expanded the list of viruses implicated in myocarditis. Initially, the molecular diagnosis of the viral etiology of myocarditis focused on the enteroviruses. More recently, parvovirus has been detected in a small proportion of heart tissues in patients with histologically documented myocarditis [9-11], although the importance of parvovirus in the pathogenesis of myocarditis, especially in adults, remains unclear.

The purpose of this report is to illustrate a rare case of serologically proven parvovirus myocarditis in which the diagnosis was confirmed by molecular techniques. In addition, the histologic features and immunophenotyping of the inflammatory infiltrates are presented.

## Case presentation

A 41-year-old African-American man with a history of hypertension presented to a health clinic with a one month history fevers, chills, mild dyspnea, generalized althralgias, and a maculopapular rash, he was treated with levofloxacin and discharged. However, five days later the patient was admitted to the emergency department with a fever of over 40°C. During the course of three days the patient had episodes of marked dyspnea, with up to forty-four breaths per minute, as well as a blood pressure of 80/50, he was intubated, placed on pressors and transferred to our institution. Further workup revealed diffuse intravascular coagulation (DIC) with D-dimer > 5000 and multiorgan failure including fulminant hepatic failure (AST 12,300 U/L, ALT 4,900), acute renal failure (BUN 52 and creatinine of 5.2), and cardio-respiratory failure. Electrocardiograms demonstrated atrial fibrillation with right bundle branch block and diffuse low voltage. A chest radiograph showed cardiomegaly and acute respiratory distress syndrome. A transthoracic echocardiogram was performed demonstrating biventricular dilatation, global hypokinesis, and right and left ejection fractions of 20% and 17%, respectively.

Serology for IgG toxoplasmosis Ab, cytomegalovirus IgM and serum DNA PCR for Parvovirus was positive. Serology was negative for human immunodeficiency virus, Epstein Barr virus, West Nile virus, viral hepatitis, Streptococcus, Influenza, Rickettsia, Coxackie, Legionella, and Adenovirus, as well as PCR for Enterovirus, Ehrlichia, and Lyme.

The patient's CD4 count was 341/ml with negative blood cultures through the hospital course, a tracheal culture taken hours before death grew Candida sp, presumably due to prolonged intubation. Despite aggressive medical support, he expired 7 days after admission.

## Methods

DNA was extracted from tissue and serum and primers were used to amplify DNA sequences of parvovirus B19 using nested polymerase chain reaction (PCR). The PCR products were separated by electrophoresis on 2% agarose gel and placed under UV light for visualization the amplified products.

DNA was extracted from tissue (Qiagen^® ^DNA kit). Four sets of primers were used to amplify DNA sequences of parvovirus B19 using nested polymerase chain reaction (PCR). PCR contained 4 different concentrations of DNA sample, 140 nmol of each primer, 200 μmol of dNTPs, 2.5 units of HotStart Taq DNA polymerase and 3.0 mmol MgCl2. DNA was amplified for 45 cycles after initial activation of HotStart Taq DNA polymerase for 15 min. The PCR products were separated by electrophoresis on 2% agarose gel and identified by ethidium-bromide staining. The gels were then placed under UV light for visualization the amplified products. The primer sequences were: PVB1 5'-AGC ATG TGG AGT GAG GGG GC-3', PVB2 5'-AAA GCA TCA GGA GCT ATA CTT CC-3'. Primers of PVB1 and 2 were used for the first round of reaction, resulting in PCR amplicon of 290 bp; a 173 bp parvovirus B19-specific amplicon was obtained by the nested PCR primers PVB3 and PVB4 (5'-GCT AAC TCT GTA ACT TGT AC-3, 5'-AAA TAT CTC CAT GGG GTT GAG-3').

Following fixation, representative sections of the right and left atria, and ventricles as well as the interventricular septum were obtained. Sections were dehydrated in serial ethanol and xylene solutions and embedded in paraffin. Six micron sections were obtained and stained withhematoxylin and eosin. Anti-Parvovirus antibody, is IgG mouse monoclonal antibody was applied to deparaffinized sections following an incubation period. The tissue was then counter stained with Hematoxylin. Finally, a bluing reagent was then applied.

### Pathological findings

An autopsy restricted to heart and lungs was performed. On external examination the patient had generalized icterus and anasarca. Internal examination revealed an unremarkable larynx and correct anatomic position of the thoracic organs. The right and left pleural cavities contained approximately 500 ml of serous fluid each. The right lung weighted 970 grams, the left 750 grams and both were severely congested, exuding a copious amount of blood and frothy fluid.

The heart weighed 460 grams, with 2 cm of aorta attached, and the pericardium removed. There was 140 ml of serous pericardial fluid. The coronary arteries arose normally, and the right coronary artery was dominant. The coronary ostia arose in the sinuses of Valsalva below the sinotubular junction. There was mild to moderate calcific atherosclerosis. The tricuspid valve and pulmonary valves were unremarkable. The right ventricular outflow and pulmonary valves were unremarkable. The mitral valve was without evidence of prolapse or annular calcification, and the papillary muscles were normal. The aortic valve was trileaflet and unremarkable. The endocardium had focal hemorrhages. The left ventricular wall measured 17 mm, the ventricular septum 4 mm, and the right ventricle 6 mm. There was no evidence of necrosis, fibrosis or infarcts. Tissue was saved and frozen for molecular studies.

**Figure 1:**
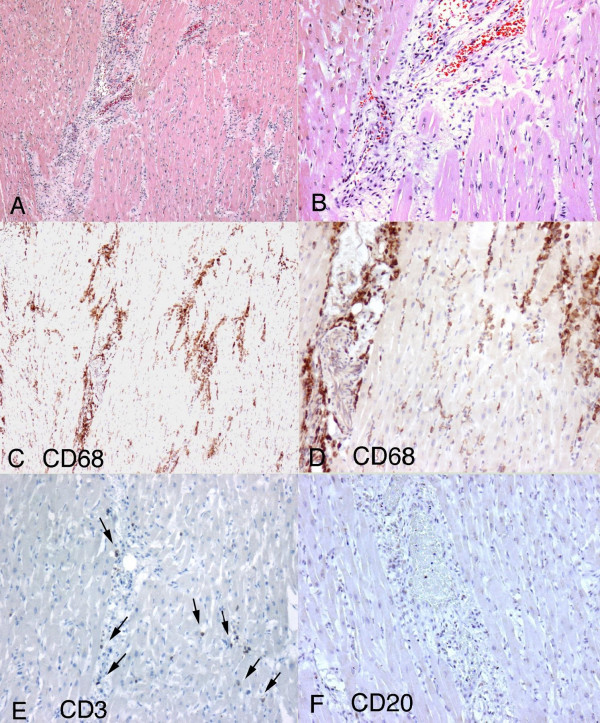
**Histopathological findings in parvoviral myocarditis**. 1A. Diffuse interstitial myocardial inflammatory infiltrate more prominent around interstitial capillaries and composed of macrophages and lymphocytes (20×). 1B. Hematoxilin-eosin stain showing vasocentric inflammation (40×). 1C-1D. CD68 positive macrophages were the most abundant cells present. (Figure 1C-10X Figure 1D-20X) 1E. Rare CD3 positive lymphocytes. 1F. Essentially negative CD20 immunohistochemical stain.

Microscopic sections of coronary arteries showed mild atherosclerosis. Sections of the left and right ventricles and ventricular septum showed no evidence of myocardial ischemia. There was a diffuse interstitial myocardial inflammatory infiltrate composed of CD68 positive macrophages (figure 1C, 1D), CD3 (figure 1E) lymphocytes in an interstitial and perivascular distribution with minimal necrosis. The infiltrates were most prominent around capillaries and arterioles (figure 1A, 1B). Immunohistochemical stains for CD20 (figure 1F) and S-100 were negative. Immunohistochemical stains for parvovirus B19 were negative as well. However, parvovirus B19 DNA was detected in cardiac muscle using non-quantitative PCR analysis. Special stains for fungi on cardiac sections were negative.

Microscopic sections of the bilateral lungs revealed diffuse alveolar damage with interstitial inflammation, but with out evidence of pneumonia or vasculitis. Immunohistochemical stains for CMV and human herpes virus were negative. A GMS stain for bacterial and fungal organisms is also negative.

## Discussion

Human parvovirus B19 infects human erythroid cells and causes hemolytic anemia, fifth disease and hydrops fetalis. [12] Other less common manifestations of the disease include acute and chronic rheumatoid-like arthropathy [13], acute glomerulonephritis [14], pneumonia and acute or chronic cardiac diseases, such as acute and chronic myocarditis, perimyocarditis, biventricular thrombi, heart failure, dilated cardiomyopathy, isolated left ventricular dysfunction and death related to cardiogenic shock [9,10,15-21]. Additionally PVB19 has been associated with myocarditis in heart transplant patients[22].

The mechanism of how parvovirus virus infects nonerythroid cells is unknown [23]. In the heart, it has been postulated that PVB19 does not infect myocytes, but endothelial cells of small intracardiac arterioles and venules, resulting in impairment of myocardial microcirculation with secondary myocyte necrosis during acute infection[24,25]. Alternatively, it has been proposed that myocarditis arises from immunological cross-reaction to epitopes shared between the virus and the myocardium [11,19].

The rate of viral genome detection in endomyocardial biopsies has been extensively explored. One study [26] reported that one third of biopsies from patients suspected to have myocarditis were positive for enteroviruses and a second one reported about one fourth[27]. Adenovirus has also been reported on detected by PCR in cardiac biopsies. Martin et al detected adenovirus DNA in 15 of 38 patients with clinical signs of myocarditis, and also found a small proportion of cases positive for cytomegalovirus and herpesvirus[28]. More recently, Grumbach et al reported enteroviruses in about 1/3 of patients with myocarditis or dilated cardiomyopathy and interestingly, did not find any case of adenovirus[29]. Calabrese found about 35% of prevalence of entero and adenoviruses in patients with myocarditis[30]. These studies did not investigate the presence of parvovirus B19. A larger series of 624 endomyocardial biopsies showed twice as much adenovirus than enterovirus by PCR [31] and only about 1% of B19 positive cases.

The rate of parvovirus detection in endomyocardial biopsies is quite variable. Schowengerdt studied 41 patients heart recipients to find a low (7%) rate of B19 genome[7]. In the last few years, additional studies showed a detection that ranged from 13 to 50% in different populations [31-35]. The detection of parvovirus by molecular methods in heart tissue is considered quite specific for symptomatic infection. In a series of 215 cardiac biopsy controls, Bowles et al did not find a single case positive for B19 genome[36].

The histologic appearance of parvoviral myocarditis has been described in a few case studies in patients with serologic or molecular confirmation of the diagnosis. There have been several reports of parvoviral myocarditis in children and immunocompromised adults. Heegaard et al reported B19 myocarditis in an adult heart transplant recipient [37]. Parvoviral infection was confirmed by IgM serology and PCR detection in myocardial tissue of viral genome. The histologic appearance was described as similar to transplant rejection, but the sample was limited to endomyocardial biopsy [38]. At least two autopsy cases have been reported of fatal parvoviral myocarditis in children. The interstitial and perivascular nature of the infiltrate and the preponderance of macrophages was stressed [11]. In one case, fatal myocarditis occurred two weeks after erythema infectiosum (Fifth's disease)[19]. The histologic features in limited cases are that of interstitial inflammation, abundant macrophages in addition to lymphocytes, rare B-cells, and patchy necrosis[19].

There have been subsequent reports of parvoviral myocarditis in immunocompetent adult patients. Bultmann described a fatal case of a 34 year-old woman with the clinical presentation of ischemic heart disease. The diagnosis of parvoviral myocarditis was made at autopsy by quantitative PCR which showed high copy numbers of viral genome is myocardial tissue[10]. Histologically, there were foci of necrosis surrounded by macrophages and T-lymphocytes, with dilatation of intramural small veins and post-capillary venules, with a vasculitis-like pattern of lymphocytic infiltration. Lamparter reported an otherwise healthy 37 year-old man who also developed symptoms of ischemic heart disease[9]. Left ventricular biopsy shows an interstitial infiltrate of T-lymphocytes and macrophages. Parvoviral genome was detected by Southern blot and PCR analysis in myocardial tissue. In adults, although the histologic features are variable, parvoviral myocarditis usually consists of non-specific interstitial perivascular mononuclear infiltrates, foci of necrosis and macrophage infiltration.

Identification of viral protein has not been accomplished in cases of parvoviral myocarditis by immunohistochemical techniques. [11,19] Possibly, there is no infection of the myocytes, and the mechanism of inflammation is immune sensitivity by cross reacting. However, one report has described detection of viral DNA in endothelial cells in a case of fatal parvoviral myocarditis[10]. In this reported case, in situ hybridization using radiolabeled antisense probes demonstrated labeling in areas of venules and arteries, suggesting infection of endothelial cells.

In addition to being responsible for a significant proportion of cases of lymphocytic myocarditis, enteroviruses have also been implicated in the transition from myocarditis to dilated cardiomyopathy (DCM)[35]. The role of parvovirus in the development of idiopathic cardiomyopathy has been recently explored. In the study published by Kuhl et al [35], 51% of the 245 patients with idiopathic left ventricular dysfunction were positive for B19 DNA against 9.4 and 1.6% of enterovirus and adenovirus respectively.

In conclusion, parvovirus should be considered as etiology in fatal myocarditis, even in adults, especially when the infiltrate is mononuclear with macrophages and interstitial predominance. Molecular techniques, specifically PCR, can establish the diagnosis post-mortem. Pre-mortem diagnosis should also be attempted, as treatment with immunoglobulin has been suggested [37] and rapid diagnosis may be necessary.

## Competing interests

The authors declare that they have no competing interests.

## Authors' contributions

FT, AB and LFGC drafted the manuscript. FT and AB evaluated the immunohistochemical stainings and confirmed the diagnoses. JSD and RZ performed the molecular studies. MTO and HQP compiled the clinical data. FT, LFGC and AB contributed to the discussion. All authors read and approved the final manuscript.

## References

[B1] Heegaard ED, Brown KE (2002). Human parvovirus B19. Clin Microbiol Rev.

[B2] Young NS, Brown KE (2004). Parvovirus B19. N Engl J Med.

[B3] Gabriel SE, Espy M, Erdman DD, Bjornsson J, Smith TF, Hunder GG (1999). The role of parvovirus B19 in the pathogenesis of giant cell arteritis: a preliminary evaluation. Arthritis Rheum.

[B4] Schwarz TF (1992). [Erythema infectiosum infection can cause intrauterine fetal death. Possibilities of diagnosis and treatment]. Fortschr Med.

[B5] Schwarz TF, Roggendorf M (1989). [Parvovirus infections in dermatology]. Z Hautkr.

[B6] Sokal EM, Melchior M, Cornu C, Vandenbroucke AT, Buts JP, Cohen BJ, Burtonboy G (1998). Acute parvovirus B19 infection associated with fulminant hepatitis of favourable prognosis in young children. Lancet.

[B7] Schowengerdt KO, Ni J, Denfield SW, Gajarski RJ, Radovancevic B, Frazier HO, Demmler GJ, Kearney D, Bricker JT, Towbin JA (1996). Diagnosis, surveillance, and epidemiologic evaluation of viral infections in pediatric cardiac transplant recipients with the use of the polymerase chain reaction. J Heart Lung Transplant.

[B8] Calabrese F, Angelini A, Carturan E, Thiene G (2006). Myocarditis and inflammatory cardiomyopathy: histomorphological diagnosis. Ernst Schering Res Found Workshop.

[B9] Lamparter S, Schoppet M, Pankuweit S, Maisch B (2003). Acute parvovirus B19 infection associated with myocarditis in an immunocompetent adult. Hum Pathol.

[B10] Bultmann BD, Klingel K, Sotlar K, Bock CT, Baba HA, Sauter M, Kandolf R (2003). Fatal parvovirus B19-associated myocarditis clinically mimicking ischemic heart disease: an endothelial cell-mediated disease. Hum Pathol.

[B11] Dettmeyer R, Kandolf R, Baasner A, Banaschak S, Eis-Hubinger AM, Madea B (2003). Fatal parvovirus B19 myocarditis in an 8-year-old boy. J Forensic Sci.

[B12] Brown KE, Anderson SM, Young NS (1993). Erythrocyte P antigen: cellular receptor for B19 parvovirus. Science.

[B13] Naides SJ (1998). Rheumatic manifestations of parvovirus B19 infection. Rheum Dis Clin North Am.

[B14] Ieiri N, Hotta O, Taguma Y (2005). Characteristics of acute glomerulonephritis associated with human parvovirus B19 infection. Clin Nephrol.

[B15] Enders G, Dotsch J, Bauer J, Nutzenadel W, Hengel H, Haffner D, Schalasta G, Searle K, Brown KE (1998). Life-threatening parvovirus B19-associated myocarditis and cardiac transplantation as possible therapy: two case reports. Clin Infect Dis.

[B16] Malm C, Fridell E, Jansson K (1993). Heart failure after parvovirus B19 infection. Lancet.

[B17] Orth T, Herr W, Spahn T, Voigtlander T, Michel D, Mertens T, Mayet WJ, Dippold W, Meyer zum Buschenfelde KH (1997). Human parvovirus B19 infection associated with severe acute perimyocarditis in a 34-year-old man. Eur Heart J.

[B18] Munro K, Croxson MC, Thomas S, Wilson NJ (2003). Three cases of myocarditis in childhood associated with human parvovirus (B19 virus). Pediatr Cardiol.

[B19] Murry CE, Jerome KR, Reichenbach DD (2001). Fatal parvovirus myocarditis in a 5-year-old girl. Hum Pathol.

[B20] Buob A, Siaplaouras S, Janzen I, Schwaab B, Hammer B, Schneider G, Kandolf R, Bohm M, Jung J (2003). Focal parvovirus B19 myocarditis in a patient with Brugada syndrome. Cardiol Rev.

[B21] Noutsias M, Kuehl U, Lassner D, Gross U, Pauschinger M, Schultheiss HP, Gutberlet M (2007). Images in cardiovascular medicine. Parvovirus-B19-associated active myocarditis with biventricular thrombi. Results of endomyocardial biopsy investigations and cardiac magnetic resonance imaging. Circulation.

[B22] Eid AJ, Brown RA, Patel R, Razonable RR (2006). Parvovirus B19 infection after transplantation: a review of 98 cases. Clin Infect Dis.

[B23] Munakata Y, Kato I, Saito T, Kodera T, Ishii KK, Sasaki T (2006). Human parvovirus B19 infection of monocytic cell line U937 and antibody-dependent enhancement. Virology.

[B24] Bultmann BD, Sotlar K, Klingel K (2004). Parvovirus B19. N Engl J Med.

[B25] Klingel K, Sauter M, Bock CT, Szalay G, Schnorr JJ, Kandolf R (2004). Molecular pathology of inflammatory cardiomyopathy. Med Microbiol Immunol (Berl).

[B26] Satoh M, Tamura G, Segawa I (1994). Enteroviral RNA in endomyocardial biopsy tissues of myocarditis and dilated cardiomyopathy. Pathol Int.

[B27] Fujioka S, Koide H, Kitaura Y, Deguchi H, Kawamura K, Hirai K (1996). Molecular detection and differentiation of enteroviruses in endomyocardial biopsies and pericardial effusions from dilated cardiomyopathy and myocarditis. Am Heart J.

[B28] Martin AB, Webber S, Fricker FJ, Jaffe R, Demmler G, Kearney D, Zhang YH, Bodurtha J, Gelb B, Ni J (1994). Acute myocarditis. Rapid diagnosis by PCR in children. Circulation.

[B29] Grumbach IM, Heim A, Pring-Akerblom P, Vonhof S, Hein WJ, Muller G, Figulla HR (1999). Adenoviruses and enteroviruses as pathogens in myocarditis and dilated cardiomyopathy. Acta Cardiol.

[B30] Calabrese F, Rigo E, Milanesi O, Boffa GM, Angelini A, Valente M, Thiene G (2002). Molecular diagnosis of myocarditis and dilated cardiomyopathy in children: clinicopathologic features and prognostic implications. Diagn Mol Pathol.

[B31] Bowles NE, Ni J, Kearney DL, Pauschinger M, Schultheiss HP, McCarthy R, Hare J, Bricker JT, Bowles KR, Towbin JA (2003). Detection of viruses in myocardial tissues by polymerase chain reaction. evidence of adenovirus as a common cause of myocarditis in children and adults. J Am Coll Cardiol.

[B32] Frustaci A, Chimenti C, Calabrese F, Pieroni M, Thiene G, Maseri A (2003). Immunosuppressive therapy for active lymphocytic myocarditis: virological and immunologic profile of responders versus nonresponders. Circulation.

[B33] Pankuweit S, Moll R, Baandrup U, Portig I, Hufnagel G, Maisch B (2003). Prevalence of the parvovirus B19 genome in endomyocardial biopsy specimens. Hum Pathol.

[B34] Klein RM, Jiang H, Niederacher D, Adams O, Du M, Horlitz M, Schley P, Marx R, Lankisch MR, Brehm MU (2004). Frequency and quantity of the parvovirus B19 genome in endomyocardial biopsies from patients with suspected myocarditis or idiopathic left ventricular dysfunction. Z Kardiol.

[B35] Kuhl U, Pauschinger M, Noutsias M, Seeberg B, Bock T, Lassner D, Poller W, Kandolf R, Schultheiss HP (2005). High prevalence of viral genomes and multiple viral infections in the myocardium of adults with "idiopathic" left ventricular dysfunction. Circulation.

[B36] Bowles NE, Ni J, Marcus F, Towbin JA (2002). The detection of cardiotropic viruses in the myocardium of patients with arrhythmogenic right ventricular dysplasia/cardiomyopathy. J Am Coll Cardiol.

[B37] Heegaard ED, Eiskjaer H, Baandrup U, Hornsleth A (1998). Parvovirus B19 infection associated with myocarditis following adult cardiac transplantation. Scand J Infect Dis.

[B38] Bultmann BD, Klingel K, Sotlar K, Bock CT, Kandolf R (2003). Parvovirus B19: a pathogen responsible for more than hematologic disorders. Virchows Arch.

